# Toxicity Profile of Combining PD-1/PD-L1 Inhibitors and Thoracic Radiotherapy in Non-Small Cell Lung Cancer: A Systematic Review

**DOI:** 10.3389/fimmu.2021.627197

**Published:** 2021-03-30

**Authors:** Butuo Li, Chao Jiang, Linlin Pang, Bing Zou, Mingjun Ding, Xindong Sun, Jinming Yu, Linlin Wang

**Affiliations:** ^1^ Department of Radiation Oncology, Shandong Cancer Hospital and Institute, Shandong First Medical University and Shandong Academy of Medical Science, Jinan, China; ^2^ Department of Otorhinolaryngology & Head and Neck Surgery, Shandong Provincial Hospital Affiliated to Shandong First Medical University, Jinan, China; ^3^ Department of Radiation Oncology, Shandong First Medical University and Shandong Academy of Medical Sciences, Tai’an, China

**Keywords:** toxicity profile, safety, immunotherapy, immune checkpoint inhibitors, thoracic radiotherapy, systemic analysis

## Abstract

**Background:**

The combination of immune checkpoint inhibitors (ICIs) and thoracic radiotherapy (TRT) has shown significant clinical activity in patients with non-small cell lung cancer (NSCLC). However, the currently available data on adverse events (AEs) were derived from a small subset of patients included in prospective clinical trials or retrospective studies. Thus, we conducted this systematic review to determine the AEs associated with this combination treatment.

**Methods:**

An electronic literature search was performed in databases and conference proceedings of prospective clinical trials assessing the combination of ICIs and TRT for patients with NSCLC. The systematic analysis was conducted to determine the profile and incidence of AEs of combination treatment. We further performed the comparison of AEs between programmed cell death 1 (PD-1) and programmed cell death ligand 1 (PD-L1) inhibitors, and sequential and concurrent administration of ICIs and TRT to help identify high risk patients. The systematic analyses were conducted with the Review Manager (version 5.3; The Cochrane Collaboration, Oxford, United Kingdom) and Stata version 12.0 (StataCorp, College Station, TX, USA) software.

**Results:**

Eleven clinical trials involving 1,113 patients with NSCLC were eligible for analysis. The incidence of all-grade AEs was 95.5%; that of high-grade AEs (grade ≥3) was 30.2%. The most frequent all-grade AE was fatigue (49.7%), while pneumonitis was the most common high-grade AE (3.8%) and grade 5 AE (0.6%). Notably, the toxicity profiles of PD-1 and PD-L1 inhibitors were similar. Concurrent treatment was associated with a higher incidence of higher-grade AEs (41.6% vs 24.8%, P=0.17) and pneumonitis (7.1% vs 3.9%, P=0.14) compared to sequential treatment, but no significant difference was observed.

**Conclusion:**

Most AEs of this combination treatment are tolerable; as the most common high-grade AE, pneumonitis deserves the utmost attention of physicians. The toxicity profiles of patients receiving PD-1 or PD-L1 were similar, and no significant difference was observed between concurrent and sequential treatment.

## Introduction

Cancer immunotherapy targets immunosuppressive molecules, such as programmed cell death 1/programmed cell death ligand 1 (PD-1/PD-L1) and cytotoxic T-lymphocyte associated antigen 4 (CTLA-4). These immune checkpoint inhibitors (ICIs) were successfully used for the treatment of patients with non-small cell lung cancer (NSCLC) of all stages and shown significant clinical activity and marked efficacy (1–3). This type of therapy has been approved by the US Food and Drug Administration for both first-and second-line treatment of metastatic NSCLC, based on significant improvements in overall response rate, progression-free survival (PFS), and overall survival (OS) (1–6). In addition, radiotherapy (RT) is also an important treatment modality for lung cancer, exerting its effects by damaging the DNA of tumor cells (7). Importantly, RT has also been recognized as an immune modulator (8). It can not only function as an “*in-situ* vaccine” by increasing the presentation of tumor-specific antigens (9), but also modulates the local tumor environment, resulting in an enhanced immune response (10).

Multiple preclinical studies have suggested a synergistic activity between ICIs and RT, by inducing the activation and recruitment of more antitumor effector T cells (11, 12), as well as the modulation of the tumor immune microenvironment (from “cold” tumor to “hot” tumor) (13–15). In addition, it has been indicated that the synergistic activity of ICIs and RT translates into prolonged survival and abscopal effect in preclinical animal models (16, 17). Furthermore, recent clinical trials also suggested the amplified antitumor effect of combination of ICIs and thoracic radiotherapy (TRT) in patients with NSCLC. The secondary analysis of 98 metastatic NSCLC patients treated with pembrolizumab in Keynote-001 trial compared patients who received previous RT with those who did not. The results revealed significantly prolonged PFS (4.4 vs. 2.1 months, respectively, P=0.019) and OS (10.7 vs. 5.3 months, respectively, P=0.026) in the former group (18). The PACIFIC trial performed the comparison of durvalumab against placebo after definitive chemoradiation for stage III NSCLC. Treatment with durvalumab was associated with significant improvements of PFS (17.2 vs 5.6 months, respectively, P<0.001) and OS (28.3 vs. 16.2 months, respectively, P<0.001) (6).

Of note, the synergistic effect of combining TRT and ICIs through modulation of the immune response may also affect the spectrum, incidence, and severity of treatment-related AEs. By targeting T cell negative feedback loops, the ICIs can impair the immune tolerance of the tumor and induce the infiltration of immune cells in normal tissues, resulting in autoimmune disease or syndromes and distinctive toxicity profiles, such as pneumonitis and thyroid dysfunction (19, 20). RT may cause a wide range of AEs through the ionizing radiation-induced DNA damage and subsequent inflammation on normal tissues, including pneumonitis, mucositis, esophagitis, fibrosis (particularly in lung tissue), and others (21).

Owing to a certain degree of overlap of the toxicity mechanism and spectrum, the combination of ICIs and TRT may exacerbate the toxicity in patients with NSCLC, particularly pneumonitis. Both the Keynote-001 and PACIFIC studies indicated a higher incidence of all-grade pneumonitis in patients who received the combination therapy. Nevertheless, the risk of developing high-grade pneumonitis did not increase significantly (6, 18). Importantly, the available evidence regarding the AEs of the combination of ICIs with TRT is limited and derived from a small subset of patients included in prospective clinical trials or retrospective studies (22).

An enhanced understanding of the spectrum and severity of toxicity would enable better prevention and management of the AEs of this combination therapy, thereby informing the clinical application and design of prospective trials. This systematic review focused on prospective clinical trials assessing the AEs of combination of ICIs with TRT in patients with NSCLC, in order to provide a complete toxicity profile and investigate the incidence of AEs of combination treatment. Notably, the treatment sequence, type of ICIs and RT was thought to have impact on the occurrence of toxicity of combination therapy. We further evaluated the role of different ICIs or treatment sequence on the incidence of AEs, so as to help identify high risk patients and guide the clinical administration of combination of ICIs and TRT.

## Materials and Methods

### Study Search and Inclusion Criteria

A comprehensive and methodical literature search was conducted to identify all prospective clinical trials investigating the combination of ICIs and TRT for patients with NSCLC. Data searches were conducted in databases, including PubMed, Embase, and the Cochrane database, from January 2000 to November 2020. Keywords included NSCLC, RT, immune checkpoint, PD-1, PD-LI, and specific ICIs drug names. Clinical trials that met the following inclusion criteria were taken into account: (1) patients with histologically confirmed NSCLC; (2) NSCLC patients receiving combination of ICIs and TRT treatment; (3) studies reporting AEs; (4) studies published in English. Retrospective studies were excluded in order to minimize the risk of bias. Abstracts and presentations were also reviewed to identify relevant clinical trials from major conference proceedings, including the American Society of Clinical Oncology, European Society of Medical Oncology, and American Society for Radiation Oncology Annual Meeting, between 2010 and 2020. The detailed information of the search strategy for the eligible studies is presented in the flow diagram according to PRISMA. All studies identified by the search strategy that met the eligibility criteria were evaluated by two independent reviewers.

### Data Extraction and Statistical Analysis

The following information was extracted from each study: National Clinical Trial number, first author, year of publication, phase of the trial, number of patients available for the analysis, age, gender, smokers, histology, line of therapy, type and dose of ICIs drugs, control groups, patterns of combination of ICIs and TRT, dose and segmentation of radiation, and number and incidence of AEs of interest (including fatigue, respiratory system, gastrointestinal tract, skin, and endocrine system toxicities). Newcastle-Ottawa-Scale (NOS) evaluation was performed to assess the quality of included studies. All data were independently reviewed and extracted by two investigators.

Some degree of heterogeneity was expected; thus, the data on AEs extracted from the studies were analyzed using DerSimonian and Laird random effect models. The inverse variance method was used to calculate the pooled incidence of AEs and their 95% confidence interval (CI). Statistical heterogeneity was evaluated with the Cochrane chi-squared test and I^2^ statistics. The publication bias was assessed by Egger’s linear regression test and funnel plots recommended by the Cochrane Collaboration. P < 0.05 was defined as significant publication bias, then non-parametric “trim-and-fill” method was performed to minimize the influence of publication bias on the results. The Z test was used to compare the AEs linked to PD-1 and PD-L1 inhibitors, as well as the sequential and concurrent administration of ICIs and TRT. All analyses were performed using the Review Manager (version 5.3; The Cochrane Collaboration, Oxford, United Kingdom) and Stata version 12.0 (StataCorp, College Station, TX, USA) software. Differences were considered statistically significant at P < 0.05.

## Results

### Characteristics of Eligible Studies

**Figure 1:**
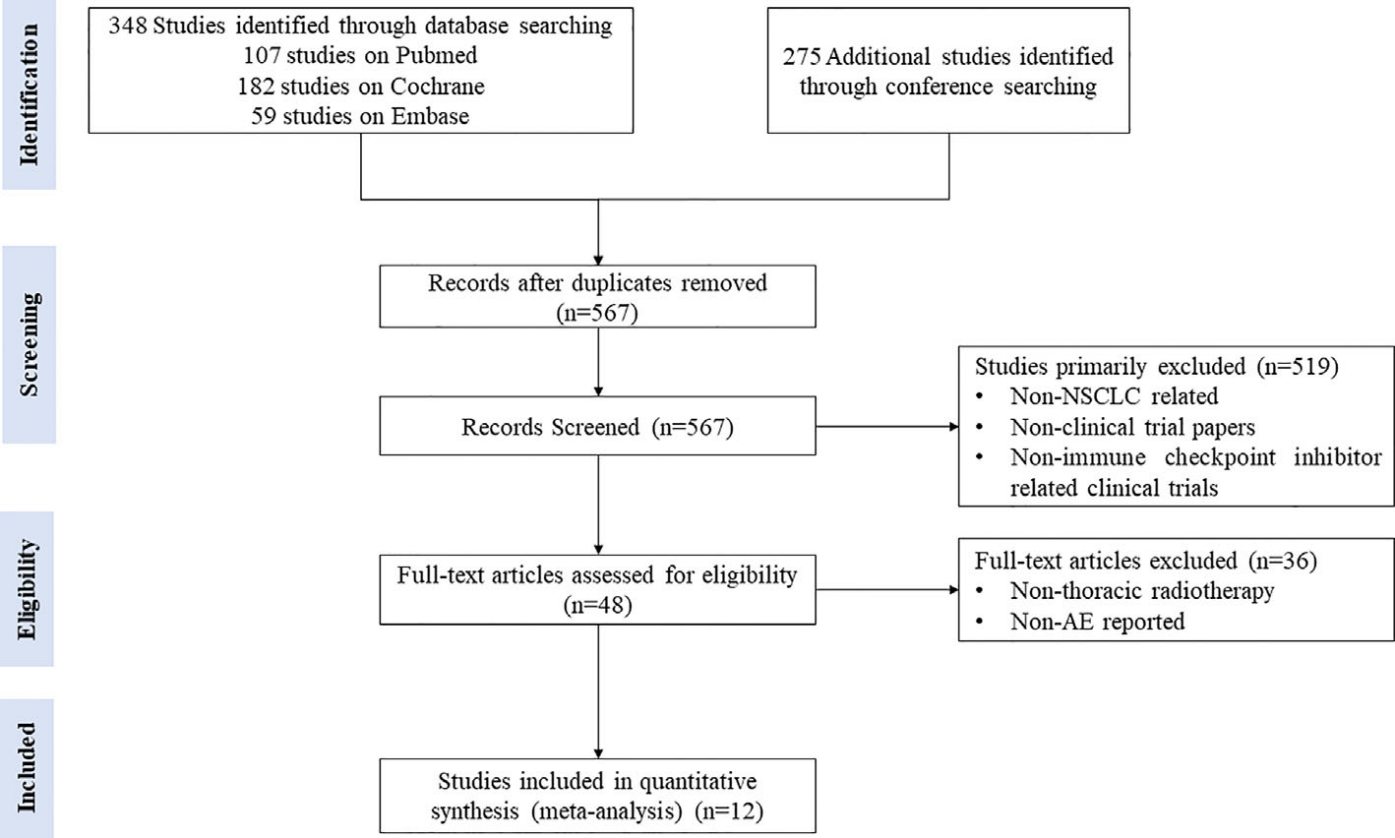
Flow diagram of study inclusion.

A total of 623 studies were retrieved and reviewed from the database searches. Of those, 56 duplicate studies were excluded. After careful screening and assessment, 11 clinical trials involving 1,113 patients with NSCLC were finally included in the analysis (6, 23–33). **Figure 1** illustrates the flow diagram of study selection.

The main characteristics of the included studies are summarized in **Table 1**. The NOS of included studies ranged from 6-8. There was 10 phase II trials and one phase III trial. Notably, PD-1 inhibitors were utilized in eight trials and PD-L1 inhibitors were utilized in three trials. Sequential administration of ICIs and RT was performed in eight trials, while concurrent therapy was performed in five trials. Patients from 9 trials received only conventional fractionated RT, while patients in one trial received only stereotactic body RT.

**Table 1:** Characteristic of clinical trials included in the analysis.

NCT number	First author	Phase	Stage of NSCLC	Median age (Range)	Gender distribution (male/female)	Smokers/non-smokers	Histology	Drugs	Line of therapy	Comparator group	Number of patients	Sequence	Radiotherapy	Newcastle-Ottawa-Scale (NOS) evaluation
NCT02125461	Antonia et al. (6)	Phase III	Unresectable III	64 (31-84)	70.2%/29.8%	91%/9%	Non-squamous 51.9%/Squamous 47.1%	Durvalumab 10mg/kg	Consolidation	Placebo	473	Sequential	Conventional 54-66Gy	8
NCT02343952	Durm et al. (23)	Phase II	Unresectable IIIA/B	66 (45-84)	64%/36%	94.6%/5.4%	Non-squamous 55%/Squamous 41%	Pembrolizumab 200mg q3w	Consolidation	None	93	Sequential	Conventional 59.4-66.6 Gy	7
NCT02434081	Peters et al. (24)	Phase II	IIIA/B	62 (41-78)	67.1%/32.9%	96.2%/3.8%	Non-squamous 59.5%/Squamous 35.4%/Missing 5.1%	Nivolumab 360mg q3w-480mg/q4w	1^st^ line	None	77	Concurrent	Conventional 66Gy/33f	8
NCT01820754	Boyer et al. (25)	Phase II	IB-IIIA	(49-75)	56%/44%	94%/6%	Adenocarcinoma 56%/Squamous 44%	Ipilimumab 10mg/kg q3w	Neo-adjuvant	None	16	Sequential	Conventional 36-60Gy	7
NCT03053856	Ahn et al. (26)	Phase II	IIIA-N2	64 (39-74)	62.2%/37.8%	N/A	Adenocarcinoma 73%/Others 27%	Pembrolizumab 200mg q3w	Neo-adjuvant	None	37	Sequential	Conventional 44Gy/22f	6
NCT03285321	Yan et al. (27)	Phase II	Unresectable IIIA/B	Arm A/B 64/62	N/A	N/A	Non-squamous 54%/Squamous 46%	Nivolumab 480mg q4w	Consolidation	Nivolumab 3mg/kg+ipilizumab 1mg/kg	50	Sequential	Conventional 59.4-66.6Gy	7
NCT02621398	Jabbour et al. (28)	Phase I/II	III	69 (53-85)	48%/52%	95%/5%	Adenocarcinoma 52%/Squamous and Others 48%	Pembrolizumab 100-200mg q3w	Consolidation	None	21	Concurrent	Conventional 60Gy/30f	6
NCT02525757	Lin et al. (29)	Phase II	IIb-IIIc	67 (50-83)	68%/32%	78%/22%	Adenocarcinoma 55%/Squamous 35%/Others 10%	Atezolizumab 1200mg q3w	Consolidation	None	40	Concurrent/sequential	Conventional 60-66 Gy/30-33f	8
NCT02444741	Welsh et al. (30)	Phase I/II	IV	N/A	65%/35%	75%/25%	Adenocarcinoma 80%/Squamous 15%/NOS 5%	Pembrolizumab 200mg q3w	Unlimited	Pembrolizumab alone	40	Concurrent	Conventional 45Gy/15f or SBRT50Gy/4f	8
NCT03631784	Jabbour et al. (31)	Phase II	Unresectable III	N/A	N/A	N/A	N/A	Pembrolizumab 200mg q3w	1^st^ line	None	185	Concurrent	Conventional 60Gy/30f	6
NCT03102242	Ross et al. (32)	Phase II	Unresectable III	63.9 (38.1-36.5)	48.4%/51.6%	61.3%/38.7%	N/A	Atezolizumab 1200mg q3w	Neo-adjuvant+ Consolidation	None	62	Sequential	Conventional 60Gy/30f	6
NCT02492568	Theelen et al. (33)	Phase II	IV	62 (35–78)	56%/44%	N/A	Nonsquamous 86%/Squamous 14%	Pembrolizumab 200mg q3w	At least 2^nd^ line	Pembrolizumab 200mg q3w alone	35	Sequential	SBRT 24Gy/3f	8

### Incidence of All-Grade AEs of Interest

The incidence of all-grade AEs in patients treated with ICIs and TRT was 95.5% (95% CI: 91.2–99.8%). The Egger’s test indicated that no significant publication bias existed except for all-grade fatigue (P=0.03). Then “trim-and-fill” analysis was conducted to addressed the bias, and fatigue was found to be the most frequent AE with the incidence of 49.7% (95% CI: 32–67.4%). AEs of the respiratory system were the second common, and the incidence of cough, dyspnea, and pneumonitis was 43.3%, 34.1%, and 23%, respectively. The funnel plots of all-grade fatigue and pneumonitis were shown in **Supplementary Figure 1**. Among those who received ICIs and TRT, nausea and diarrhea (AEs related to the gastrointestinal tract) occurred in 29.1% and 15.8% of patients, respectively. Of note, the incidence of pruritus, dermatitis, rash, and thyroiditis was 12.4%, 11.2%, 13.4%, and 9.4%, respectively (**Table 2**).

**Table 2:** Incidence of all-grade AEs of interest.

	Incidence	95%CI	Heterogeneity	χ^2^	Egger test P
All-grade AEs	95.5%	91.2%-99.8%	64.1%	5.56	0.48
Fatigue	49.7%	32%-67.4%	95.2%	126.3	0.03
Cough	43.3%	25.2%-61.5%	96.5%	170.1	0.46
Dyspnea	34.1%	21.8%-46.4%	91.6%	71.49	0.17
Pneumonitis	23%	14.2%-31.7%	85.6%	48.4	0.27
Nausea	29.1%	15.8%-42.5%	94.8%	115.8	0.13
Diarrhea	15.8%	9.8%-21.7%	72.1%	17.9	0.89
Rash	13.4%	9.4%-17.5%	37.6%	8.02	0.40
Dermatitis	11.2%	0%-22.6%	85.3%	13.6	0.11
Pruritus	12.4%	9.4%-15.3%	14.7%	4.69	0.63
Thyroiditis	9.4%	3.3%-15.4%	90.3%	61.7	0.1

### Incidence of High-Grade AEs of Interest

**Table 3** represents the incidence of high-grade (grade ≥3) AEs in patients treated with ICIs and TRT. The funnel plots of high-grade AEs and pneumonitis were shown in **Supplementary Figure 1**. The Egger’s test indicated the publication bias of high-grade fatigue, dyspnea, pneumonitis, nausea, colitis, and rash, and “trim-and-fill” analysis was conducted to address the bias and calculate the pooled incidence. The incidence of high-grade AEs among all patients was 30.2% (95% CI: 18.2–42.1%). Pneumonitis was the most common high-grade AE (3.8%, 95% CI: 2.0–6.9%), followed by dyspnea (2.1%) and colitis (0.5%). Besides, the incidence of high-grade fatigue, cough, nausea and colitis was 0.3%, 0.3%, 0.1% and 0.5%, respectively. Notably, the incidence of grade 5 AEs was 1.5% (95% CI: 0–3.1%), and pneumonitis also exhibited the highest incidence (0.6%, 95% CI: 0.1–1.1%).

**Table 3:** Incidence of high-grade AEs of interest.

	Incidence	95%CI	Heterogeneity	χ^2^	Egger test P
High-grade AEs	30.2%	18.2%-42.1%	93.5%	122.2	0.97
Fatigue	0.3%	0-1.8%	52.9%	25.9	0.02
Cough	0.3%	0-0.8%	0.0	0.85	0.67
Dyspnea	2.1%	0%-4.2%	36.3%	16.4	0.008
Pneumonitis	3.8%	1.1%-6.6%	80.1%	52.2	0.009
Nausea	0.1%	0%-0.3%	0.0	0.43	0.005
Colitis	0.5%	0-1.3%	0.0	2.34	0.016
Rash	0.3%	0-0.8%	0.0	6.19	0.038
Grade 5 AEs	1.5%	0%-3.1%	70.8%	20.5	0.20
Pneumonitis	0.6%	0.1%-1.1%	0.0	5.21	0.16

### Difference in the Incidence of AEs Between PD-1 and PD-L1 Inhibitors Combined With TRT

The comparison of AEs between PD-1 and PD-L1 inhibitors combined with TRT is shown in **Table 4**. In terms of all-grade AEs, fatigue was the most common in the PD-1 inhibitor group and showed a similar incidence to that observed in the PD-L1 inhibitor group (50.2% vs. 49%, respectively, P=0.97). All-grade cough was most frequent in patients with PD-L1 inhibitors and TRT; however, there was no significant difference observed compared with PD-1 inhibitors (60% vs. 36.6%, respectively, P=0.39). Notably, the incidence of pneumonitis was comparable between PD-1 and PD-L1 inhibitors combined with TRT (20.7% vs. 30%, respectively, P=0.21). Furthermore, we did not find significant differences in the incidence of other AEs.

**Table 4:** Difference in incidence of AEs with PD-1 vs PD-L1 inhibitors combined with thoracic radiotherapy.

	PD-1	PD-L1	P
**All-grade AEs**			
Fatigue	50.2% (32.2%-68.2%)	49% (0%-99.1%)	0.97
Cough	36.6% (14.5%-58.8%)	60% (11.5%-99%)	0.39
Dyspnea	30.6% (15.3%-46%)	44.4% (0.2%-88.6%)	0.56
Pneumonitis	20.7% (11%-30.5%)	30% (19.1%-40.8%)	0.21
Nausea	28.2% (8.2%-48.2%)	33.8% (0%-74%)	0.81
Diarrhea	13.3% (5.5%-21.1%)	18.8% (13.2%-20.8%)	0.20
Thyroiditis	7.3% (1.1%-13.6%)	11.8% (9%-14.6%)	0.20
Rash	12.4% (7.5%-17.3%)	18.3% (3.7%-32.9%)	0.45
Pruritus	12.6% (7.3%-17.8%)	12.5% (9.5%-15.5%)	0.97
**High-grade AEs**			
Grade≥3 AEs	25.6% (5.8%-45.4%)	36.6% (19.3%-53.8%)	0.25
Fatigue	1.5% (0%-3.2%)	5.2% (0-17.1%)	0.542
Cough	0.5% (0-1.3%)	0.4% (0-0.9%)	0.85
Dyspnea	3.7% (0.7-6.6%)	3.1% (0%-8.3%)	0.84
Pneumonitis	6% (2.4%-9.6%)	3.3% (1.7%-4.8%)	0.18
Rash	1.6% (0-3.4%)	0.3% (0-0.7%)	0.32

Moreover, the incidence of high-grade AEs was similar between the PD-1 and PD-L1 inhibitor groups (25.6% vs. 36.6%, respectively, P=0.25). Pneumonitis was the most common high-grade AE in the PD-1 inhibitor group, and there was no significant difference observed compared with the PD-L1 inhibitor group (6% vs. 3.3%, respectively, P=0.18). Besides, there was also no significant difference in the incidence of high-grade fatigue, cough, dyspnea, and rash. In summary, no significant difference of the incidence of AEs was observed in the PD-1 or PD-L1 inhibitors when combined with TRT.

### Difference in the Incidence of AEs Between Concurrent and Sequential Administration of ICIs and TRT

**Table 5** describes the comparison of the toxicity profile between the concurrent and sequential administration of ICIs and TRT. In terms of all-grade AEs, fatigue was the most common in both groups, and there was no significant difference observed between sequential and concurrent treatment (45.5% vs. 57.3%, respectively, P=0.49). Compared with patients receiving sequential ICIs and TRT, those who received concurrent treatment had a slightly higher incidence of all-grade pneumonitis; however, this difference was not statistically significant (25.8% vs. 21.3%%, respectively, P=0.66). Although no significant difference was observed, the incidence of other respiratory AEs, including cough and dyspnea, was also higher in concurrent treatment group. Moreover, there were also no significant differences in the incidence of all-grade nausea, thyroiditis, and pruritus between concurrent and sequential ICIs and RT.

**Table 5:** Difference in incidence of AEs with concurrent vs sequential ICIs and thoracic radiotherapy.

	Sequential	Concurrent	P
**All-grade AEs**			
Fatigue	45.5% (26.2%-54.8%)	57.3% (35.1%-68.7%)	0.49
Cough	44.5% (26.8%-62.2%)	51.9% (13.7%-90.1%)	0.73
Dyspnea	24.5% (17.5%-31.6%)	45% (16.5%-73.4%)	0.17
Pneumonitis	21.3% (10.1%-32.5%)	25.8% (9.3%-42.2%)	0.66
Nausea	16.4% (9.1%-23.8%)	41.9% (10.2%-73.6%)	0.13
Diarrhea	18.2% (14.2%-22.2%)	14.1% (5%-23.3%)	0.42
Thyroiditis	10.2% (7.3%-13.2%)	9% (0%-21.4.4%)	0.84
Pruritus	13.5% (8.9%-18%)	10.5% (9.5%-15.3%)	0.42
**High-grade AEs**			
Grade≥3 AEs	24.8% (13.1%-36.5%)	41.6% (22.1%-61%)	0.17
Fatigue	1.9% (0-4.7%)	1.6% (0-4.2%)	0.89
Cough	0.5% (0-1%)	0.4% (0-1.4%)	0.93
Dyspnea	4.3% (0.2%-8.5%)	2.1% (0-4.2%)	0.35
Pneumonitis	3.9% (0.7%-7.1%)	7.1% (4.4%-9.7%)	0.14
Nausea	1.3% (0-3.1%)	0.7% (0-2%)	0.62
Colitis	0.8% (0-2.0%)	0.5% (0-1.9%)	0.80

Concurrent treatment with ICIs and TRT was related to a slightly higher incidence of high-grade AEs compared with sequential treatment (41.6% vs. 24.8%, respectively, P=0.09). Although no significant difference was observed, concurrent ICIs and TRT was associated with higher rate of high-grade pneumonitis compared to sequential treatment (7.1% vs 3.9%, P=0.14). Besides, the risk of high-grade fatigue, cough, dyspnea, nausea, and colitis was also similar between the concurrent and sequential treatment groups. In summary, the incidence of AEs of patients receiving concurrent ICIs and TRT was comparable to sequential treatment.

## Discussion

The potential synergistic effect of the combination of ICIs and TRT has been reported in several preclinical studies (11–15). According to prospective clinical trials, this effect translates into survival benefit for patients with NSCLC (1, 4–6). However, the currently available safety information is primarily based on a limited set of studies. Thus, the present study is the first to systematically characterize the toxicity profiles and demonstrate the safety and tolerability of the combination of ICIs and TRT in patients with NSCLC.

The efficacy of combining TRT and immunotherapy is believed that 1 + 1 equal more than 2 (15), whether the synthetic effect of combination treatment would double the toxicities remains to be clarified. The potential mechanisms involved in the toxicity associated with this combination treatment are unknown. While both ICIs and TRT have the capacity to evoke toxicities in normal tissues when administered alone, and the synthetic effect may also induce the overlap of the profile and mechanism of toxicity (34, 35).

The underlying etiology and mechanisms of AEs associated with ICIs is suggested to be related to the disruption of immunologic homeostasis (36). This results in an immune-boosting effect through a series of processes involving autoreactive lymphocytes, autoantibodies, and cytokines (37, 38). The AEs associated with ICIs are the consequences of excessive immunity against normal tissue, involving autoimmune and pro-inflammatory manifestations in the skin, endocrine, gastrointestinal, respiratory, and cardiovascular systems, etc. (36). In addition, TRT may also cause a wide range of AEs, including pneumonitis, mucositis, esophagitis, fibrosis (particularly in lung tissue), and others (39, 40), which is suggested to be induced by the induction of DNA breaks, production of reactive oxygen species (41), and the release of damage-associated molecular patterns (DAMPs) (42, 43). These effects lead to subsequent acute inflammation-like pneumonitis, mucositis, and esophagitis in the short term (44, 45). And succeeding repair and regeneration processes could manifest as chronic events to drive excessive tissue remodeling, resulting in late-onset toxicity such as fibrosis (45–47). The immunological response and altered microenvironment play a central role in the development of either short- or long- term toxicity related to TRT.

The administration of ICIs could also magnify the inflammatory response in irradiated normal tissue and result in infiltration of redundant immunocytes infiltrating and release of inflammatory factors. Furthermore, there may be a certain degree of overlap between the toxicities of RT and immunotherapy. In theory, the combination of TRT and ICIs should be associated with increased toxicity in patients with NSCLC; yet, the degree of increase remains unclear.

We performed this systematic analysis of 1,113 patients with NSCLC who received treatment with the combination of ICIs and TRT in 11 prospective clinical trials; the incidence of all-grade AEs was 95.5%, while that of high-grade AEs was 30.2%. These rates are higher than those of AEs caused by ICIs monotherapy in a previous meta-analysis (65.8% and 16.5%, respectively) (48). As expected, the combination of ICIs and TRT was associated with higher toxicity; however, the observed increase remained within acceptable levels. Even so, stricter screening prior to initiating treatment and closer monitoring during treatment should be performed for NSCLC patients receiving combination of TRT and ICIs, which would help to decrease the incidence of AE and avoid fatal AE.

Similar to treatment with ICIs, the combination of ICIs and TRT also results in a wide variety of AEs, including fatigue, skin toxicity, and events related to the respiratory system, gastrointestinal tract, and endocrine system. Fatigue was the most frequent among all-grade AEs in patients with NSCLC treated with the combination of ICIs and TRT, which was consistent with the toxicity profile of ICIs (49, 50). Besides, colitis, thyroiditis and hepatitis are the common autoimmune disease of ICIs, mediated by cytotoxic T cells against corresponding organs. Our systemic review found that the incidence of high-grade colitis in NSCLC patients receiving ICIs and TRT was 0.5%, which was similar to the high-grade colitis caused by ICIs monotherapy (0.6%) (51). And thyroiditis was also well-tolerated with the incidence of 9.4%. Whereas only 2 studies reported the incidence of hepatitis among 11 studies. Ahn et al. reported one cases of grade 3 autoimmune hepatitis among 37 patients (8.1%) (26), and Theelen et al. reported that none of 35 patients developed hepatitis (33). The combination of ICIs and TRT didn’t significantly expand the incidence of hepatitis compared to ICIs monotherapy (5-10%) (52). In summary, the outer-pulmonary toxicity of ICIs and TRT was well-tolerated, and was not significantly elevated compared to ICIs monotherapy. Just as ICIs monotherapy, regular measurement of thyroid and liver function is also required during combination treatment. Besides, the occurrence of diarrhea should be alert to colitis, whose symptom may not correlate with colitis severity as seen by endoscopy and histology (53).

The potential mechanisms of outer-pulmonary AEs induced by ICIs may include the similar antigenic epitope and cross-reactivity of T cells against tumor and normal tissue, and elevated cytokines (36). With the joint of RT, the “*in-situ* vaccination and immunomodulation effect” leads to the increased release of antigen and elevated infiltration of lymphocytes. Then the similar antigenic epitope in normal tissue induced the elevated recruitment of immune cells, and release of cytokines and antibodies, followed by excessive immunity against normal tissue, involving autoimmune and pro-inflammatory manifestations. Due to the limited amount of specific antigen in outer-pulmonary tissues, there was no significant increase of infiltrated immune cells and excessive immunity on normal tissues. Thus, only a slightly increase of related outer-pulmonary AEs was observed in this systemic review, with an incidence of high-grade AEs less than 3%.

However, the cumulative toxicity of radiation and ICIs could give rise to the higher incidence of inter-pulmonary AEs. The incidence of all-grade cough, dyspnea and pneumonitis was 43.3%, 34.1% and 23%. Thus, the pulmonary function test and routine CT scans prior to initial treatment are recommended to guide the patterns of combination treatment, such as the dose and fraction of TRT. Pneumonitis was the most common among high-grade AEs (3.8%) and grade 5 AEs (0.6%), which is higher than that of ICIs monotherapy (52), and associated with increased treatment discontinuation and mortality in NSCLC patients treated with combination therapy (6, 26). CT scans should also be performed in the process of treatment to evaluate pneumonitis, and early detection and timely intervention (such as dose adjustment) could decrease the rate of discontinuation of treatment and treatment-related death to a great extent.

The TRT-induced DNA damage contributes to the injury of lung tissue, and is followed by the release of antigen and inflammatory factors (e.g., tumor necrosis factor [TNF] and transforming growth factor beta [TGF-β]). And the administration of ICIs unleashes T cells to kill the tumor and repair normal tissue. Moreover, the recruitment of redundant immunocytes in lung tissue may magnify inflammation and exacerbate the pulmonary toxicity (54). Previous pre-clinical studies also showed that changes in inflammatory and a 2.1-fold increase of CD8+ T-cells were observed in irradiated lung tissues of mice receiving RT and ICIs compared with RT alone; however, there was no significant elevation in mortality (55). And elevated TNF, which mediates the synergistic effect of the combination treatment, was also associated with pulmonary toxicity (13, 56).

In addition, exposure to smoking and poor condition of the lung due to other diseases (e.g., obstructive pulmonary disease) are related to increased toxicity in patients with NSCLC (57). Also, the presence of tumor burden in the lung may limit the tolerance to injury. Thus, it is recommended that clinicians carefully evaluate the risk of pneumonitis based on the smoking history, pulmonary function test, and others, and allocate more of their attention to prevent, monitor, recognize, and manage pneumonitis at the early stage of treatment with ICIs and TRT. Thorough understanding of the mechanism of toxicity caused by the combination treatment is urgently needed to determine useful biomarkers for the identification of high-risk patients.

The exploration of related factors of toxicity could help to identify high risk patients and enable better prevention and management of the AEs of combination of TRT and ICIs. And the parameters of ICIs drugs or radiotherapy, and sequence of treatment were thought to play important roles on the AEs of combination treatment. At present, there is no head-to-head study to compare the difference in AEs between PD-1 and PD-L1 inhibitors combined with TRT. A previous study stated that the toxicity profiles of PD-1 and PD-L1 inhibitors in NSCLC patients are similar (58). And PD-1 inhibitors have been associated with a significantly higher incidence of high-grade immune-related pneumonitis (1.1% vs 0.4%, P=0.01) (59). The potential mechanism involved in the higher incidence of pneumonitis may be the blockage of PD-1-PD-L2 induced by PD-1 inhibitors. This blockage assists in the release of cytokines and proliferation of self-reactive T cells, leading to the enhancement of the antitumor effect and AEs (60).

When combined with TRT, no significant difference was recorded between PD-1 and PD-L1 inhibitor in our systemic review. Notably, the combination of TRT and PD-1 or PD-L1 inhibitors were related to higher incidence of pneumonitis compared to PD-1 or PD-L1 monotherapy. Both ICIs and TRT participated in the development and progression of pneumonitis, and TRT predominated on account of the DNA damage, subsequent inflammatory response, and collagen deposition on normal lung tissue. The leading role of TRT rather than ICIs might be the reason for the similar incidence of PD-1 and PD-L1 inhibitors when combined with TRT. Thus, the selection of candidate ICIs is recommended, primarily depending on their efficacy rather than the toxicity.

Moreover, the role of treatment sequence of TRT and ICIs on the incidence of toxicity was of close concern. Due to the time-dependent effect induced by TRT in normal tissue, the toxicity ranges from acute inflammatory effects towards chronic fibrotic side effects (45, 61, 62). Thus, concurrent or sequential treatment with ICIs and TRT may induce different side effects, particularly in lung tissue. Concurrent treatment is theoretically associated with higher toxicity due to the acute phase inflammation and overlapping toxicity. However, the collective available evidence on the safety of concurrent or sequential treatment with ICIs and TRT is varied. The secondary analysis of the PACIFIC study revealed that patients with NSCLC who received durvalumab within 14 days from the last session of TRT had superior survival and a higher rate of pneumonitis (63). Nevertheless, another retrospective study of 79 patients did not find differences in AEs between the concurrent and sequential administration of ICIs and RT (22).

As expected, this systematic analysis revealed that concurrent administration of ICIs and TRT led to an improved toxicity profile, particularly with regard to pneumonitis; while no statistical significance was found. In addition, the increase of outer-pulmonary AEs by concurrent treatment was not obvious. Thus, pulmonary function test and routine CT scans are essential for NSCLC patients receiving concurrent ICIs and TRT. The potential mechanisms for the statistically undifferentiated incidence of AEs between the concurrent and sequential treatments are unknown, and a hypothesis is provided below. Firstly, previous evidence has demonstrated the “long tail effect” of ICIs on the survival of patients with NSCLC (64–66). While the “long tail effect” and immunological memory of ICIs could also give rise to long-lasting AEs, which may contribute to the increased incidence of AEs when combined with subsequent RT. Moreover, immunotherapy followed by RT was also found to induce radiation recall pneumonitis, which was triggered by a “remembered” and “overreacted” process of the immunomodulatory effect (67). Thus, sequential treatment could not completely avoid the overlapping toxicity and significantly decrease the occurrence of AEs, as initially envisioned. Further studies are warranted to identify the acute and long-term toxicity, as well as the respective mechanisms of different sequences of RT and ICIs combination therapy.

Except for the sequence of treatment, the dose and fraction of TRT were also associated with the toxicity of TRT and ICIs. Welsh et al. performed an exploratory analysis and revealed that the median PFS was better in SBRT group compared to traditional RT (20.8 vs 6.8 months, P=0.03) in metastatic NSCLC patients, and 3 and 5 patients experienced high-grade AEs in SBRT and traditional RT group respectively. Based on available data, there was no significant difference on toxicity between two groups (30). However, further studies are needed to assess the difference on efficacy between SBRT and conventional radiotherapy combined with ICIs, especially for metastatic NSCLC patients. In addition, the radiotherapy dose and site for metastatic NSCLC should also be taken into consideration in future studies to evaluate the safety and efficacy of combining ICIs and TRT.

A limitation of this study is the relatively small number of eligible studies included in our analyses. However, all published clinical trials of the combination of ICIs and TRT in patients with NSCLC were included to capture the safety data. In addition, the assessment of AEs was somewhat subjective and varied between studies. Thus, our analysis depended on the quality of AE reporting by investigators. Moreover, there was heterogeneity among the studies included in this systematic analysis. Further larger scale, multicenter, randomized controlled trials and real-word studies are warranted to evaluate the safety of the combination of ICIs and TRT in patients with NSCLC.

## Conclusion

This systematic review, for the first time, draws attention to the toxicity profile of the combination of ICIs and TRT for patients with NSCLC, and focused comprehensive effort at the comparison of AEs based on different ICIs and different treatment settings. Most AEs of the combination treatment are tolerable. Nonetheless, pneumonitis was the most common high-grade AE and deserves the utmost attention of physicians due to its leading role in AE-related death. Careful selection of patients at high risk and close monitoring for pneumonitis in patients with NSCLC receiving the combination of ICIs and TRT are recommended. This systematic analysis also demonstrated similar safety profiles between PD-1 and PD-L1 inhibitors combined with TRT, and a relatively higher incidence of AEs induced by concurrent treatment. Above all, optimal treatment selection is recommended, primarily depending on the efficacy rather than the safety of the candidate drugs. Furthermore, the identification of patients at high risk of toxicity is necessary prior to the administration of concurrent ICIs and RT. The findings of this comprehensive analysis could lay a foundation to accelerate the development of ICIs and TRT combination treatment, and achieve the goal of maximizing benefit and minimizing toxicity.

## Data Availability Statement

The raw data supporting the conclusions of this article will be made available by the authors, without undue reservation.

## Author Contributions

Methodology, BL, CJ, LP, and BZ. Software, BL and MD. Validation, XS, JY, and LW. Formal analysis, BL, CJ, BZ, and LW. Data curation, BL, LP, BZ, MD, and XS. Writing–original draft preparation, BL, CJ, LP, and LW. Writing–review and editing, BL, CJ, XS, and LW. Visualization, MD, BZ, JY, and XS. Project administration, XS, JY, and LW. Funding acquisition, JY and LW. All authors contributed to the article and approved the submitted version.

## Funding

This work was supported by the following grant: Natural Science Foundation of Shandong Province (Grant No. ZR2019LZL012), the Innovation Project of Shandong Academy of Medical Sciences (2019-04), and the Academic Promotion Program of Shandong First Medical University (2019ZL002).

## Conflict of Interest

The authors declare that the research was conducted in the absence of any commercial or financial relationships that could be construed as a potential conflict of interest.

## Supplementary Material

Supplementary Figure 1 Funnel plots for **(A)**. All-grade fatigue, **(B)**. All-grade pneumonitis, **(C)**. High-grade AEs, **(D)**. High-grade pneumonitis. Due to the publication bias of all-grade fatigue and high-grade pneumonitis, the figure presents the funnel plot with the missing studies imputed by the trim-and-fill method.
