# Supplementary figures and images for: Toxicity Profile of Combining PD-1/PD-L1 Inhibitors and Thoracic Radiotherapy in Non-Small Cell Lung Cancer: A Systematic Review
Source: Front Immunol. 2021 Mar 30;12:627197. doi: 10.3389/fimmu.2021.627197 (PMC8042254; doi:10.3389/fimmu.2021.627197)

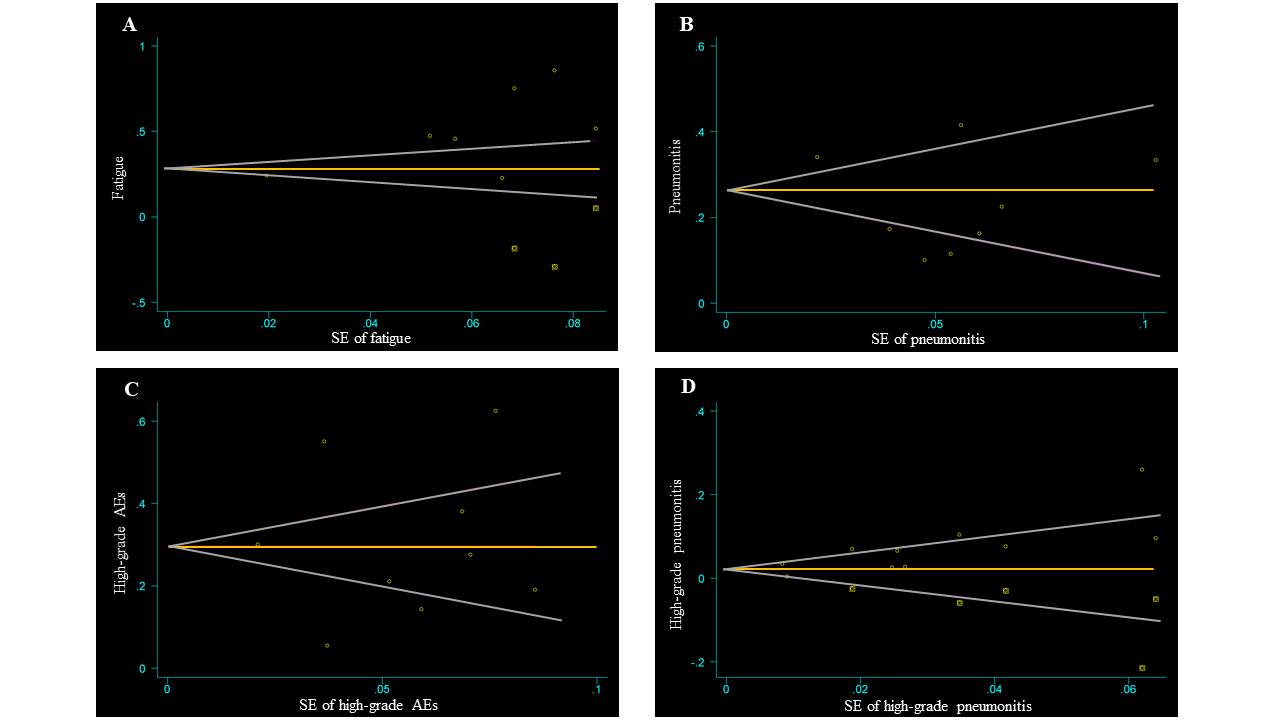

Supplement: Supplementary Figure 1 — Funnel plots for (A). All-grade fatigue, (B). All-grade pneumonitis, (C). High-grade AEs, (D). High-grade pneumonitis. Due to the publication bias of all-grade fatigue and high-grade pneumonitis, the figure presents the funnel plot with the missing studies imputed by the trim-and-fill method. [file Image_1.tif]
